# Comparative analysis of left bundle branch area pacing in patients with and without a history of open‐heart surgery

**DOI:** 10.1002/joa3.70010

**Published:** 2025-01-30

**Authors:** Yasumasa Nohno, Ryosuke Kozu, Kii Ito, Yuta Chikazawa, Shusaku Maruyama, Tomoya Hasegawa, Hiromi Tsuchiya, Takahiro Tachibana, Hikaru Kimura, Yoshikazu Yazaki

**Affiliations:** ^1^ Department of Cardiology Saku Central Hospital Advanced Care Center Saku Japan; ^2^ Department of Cardiology Asama Nanroku Komoro Medical Center Komoro Japan

**Keywords:** cardiac surgical procedures, conduction system pacing, left bundle branch area pacing, lumenless lead, permanent pacemaker implantation

## Abstract

**Background:**

Left bundle branch area pacing (LBBAP) is widely performed in routine clinical practice. Achieving LBBAP requires deep insertion of the lead into the interventricular septum. LBBAP may be challenging in patients with a history of open‐heart surgery (OHS) because of myocardial fibrosis associated with surgical trauma. This study aimed to report the feasibility and safety of performing LBBAP in patients with a history of OHS.

**Methods:**

This retrospective analysis included patients who underwent successful LBBAP between November 2020 and September 2024, with approval from our institutional review board. LBBAP was performed using a 3830 SelectSecure lead, and pacing parameters were assessed before and after implantation.

**Results:**

One hundred patients were analyzed, including 26 in the OHS group and 74 in the non‐OHS group. The success rates of LBBAP were 84.6% in the OHS group and 90.5% in the non‐OHS group (*p =* 0.375). Notably, the number of LBBAP lead placements was higher in the OHS group (3.0 ± 2.1 vs. 2.0 ± 1.4, *p =* 0.017). The left ventricular activation time in lead V6 was comparable between the groups at implantation (73.6 ± 13.3 ms vs. 75.6 ± 12.1 ms, *p =* 0.522). The QRS duration was significantly wider in the OHS group at implantation (131.3 ± 14.6 vs. 121.1 ± 12.3 ms, *p =* 0.002), but parameters remained stable at 1 year.

**Conclusions:**

LBBAP in patients with a history of OHS may present a slightly higher level of technical difficulty, but it is both feasible and safe.

## Graphical abstract

This study highlights the feasibility and safety of left bundle branch area pacing (LBBAP) in patients with a history of open‐heart surgery (OHS). Despite increased technical difficulty, LBBAP achieved comparable success rates in OHS and non‐OHS groups, with stable pacing parameters observed over 1 year.

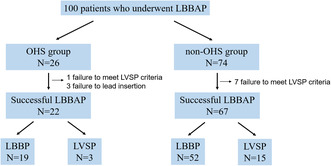

## 1 INTRODUCTION

Cardiac pacing is an established therapy for bradyarrhythmias. Generally, the ventricular lead is placed at the right ventricular (RV) apex or septum. However, chronic RV pacing causes left ventricular (LV) dyssynchrony, leading to LV dysfunction and heart failure.^1^

One management strategy for the deleterious effect of RV pacing involves upgrading to biventricular cardiac resynchronization therapy (CRT). Recently, conduction system pacing (CSP), including His‐bundle pacing (HBP) and left bundle branch area pacing (LBBAP), has emerged as a form of CRT, and several studies have reported its efficacy in this regard.^2^, ^3^ HBP is the best approach for physiological pacing;^4^ however, it has limitations, including a longer procedure and fluoroscopy time, lower R‐wave amplitude, and a higher threshold than LBBAP.^5^ Therefore, LBBAP is widely used in routine clinical practice.

Achieving LBBAP requires deep insertion of the lead into the interventricular septum. LBBAP may be challenging in patients with a history of open‐heart surgery (OHS) because of myocardial fibrosis associated with surgical trauma. However, the feasibility and success rate of LBBAP in patients with a history of OHS are not well described.

This study thus investigated the feasibility and safety of LBBAP in patients with a history of OHS.

## 2 METHODS

### 2.1 Patient selection

Consecutive patients with successful LBBAP were enrolled between November 2020 and September 2024. The study protocol was approved by the Institutional Review Board of Saku Central Hospital (R202410‐01) and all patients provided written informed consent. The research reported in this study adhered to the guidelines of the Declaration of Helsinki (revised in 2013).

### 2.2 Procedure details

LBBAP was performed using a 3830 SelectSecure lead and a C315HIS‐sheath (Medtronic Inc., Minneapolis, MN, USA). Twelve‐lead surface electrocardiography (ECG) and intracardiac pacing were performed. The sheath with the pacing lead was placed at the mid‐septum of the RV and screwed deep into the ventricular septum. The lead depth was evaluated using angiographic lead motion and detection of premature ventricular contraction during screw placement.^6^, ^7^

### 2.3 Definition of left bundle branch capture

LBBAP includes left bundle branch pacing (LBBP) and LV septal pacing (LVSP). Successful LBBP was defined as a Qr or qR pattern in lead V1 with unipolar pacing in addition to any one of the following left bundle branch (LBB) capture criteria: (1) short and constant LV activation time (LVAT) in lead V6 of <80 ms, (2) a V_6_‐V_1_ interpeak interval of >44 ms, (3) demonstrated LBB potential, (4) a change in QRS duration and morphology when performing threshold testing or programmed stimulation. LVSP was defined in cases where the Qr or qR pattern was demonstrated in lead V1 with unipolar pacing, but did not meet the aforementioned criteria.

As a principle, LBBP was attempted in all cases. Even if LVSP was achieved during the initial lead placement, additional screwing was performed to pursue LBBP. In cases where achieving LBBP was challenging, the decision on the number of attempts to make was left to the discretion of the operator.

### 2.4 Data collection and follow‐up

Baseline data obtained included patient demographic information, echocardiographic data, electrograms recorded from pacing leads, pacing parameters, and 12‐lead ECGs.

After device implantation, pacing parameters and 12‐lead ECG were recorded at discharge (days 1–7 post‐operation) and at regular follow‐ups conducted every 6 months.

### 2.5 Statistical analyses

The pacing parameter values (capture threshold, R‐wave amplitude, and impedance) and QRS duration were presented as means and standard deviations. Categorical variables were presented as numbers and percentages. Unpaired *t*‐tests were used for comparisons among groups, and Fisher's exact test was used for comparisons between groups. Statistical significance was set at *p* < 0.05 was considered significant. Statistical analyses were performed using EZR version 1.61 (Saitama Medical Center, Jichi Medical University, Saitama, Japan).

## 3 RESULTS

**FIGURE 1:**
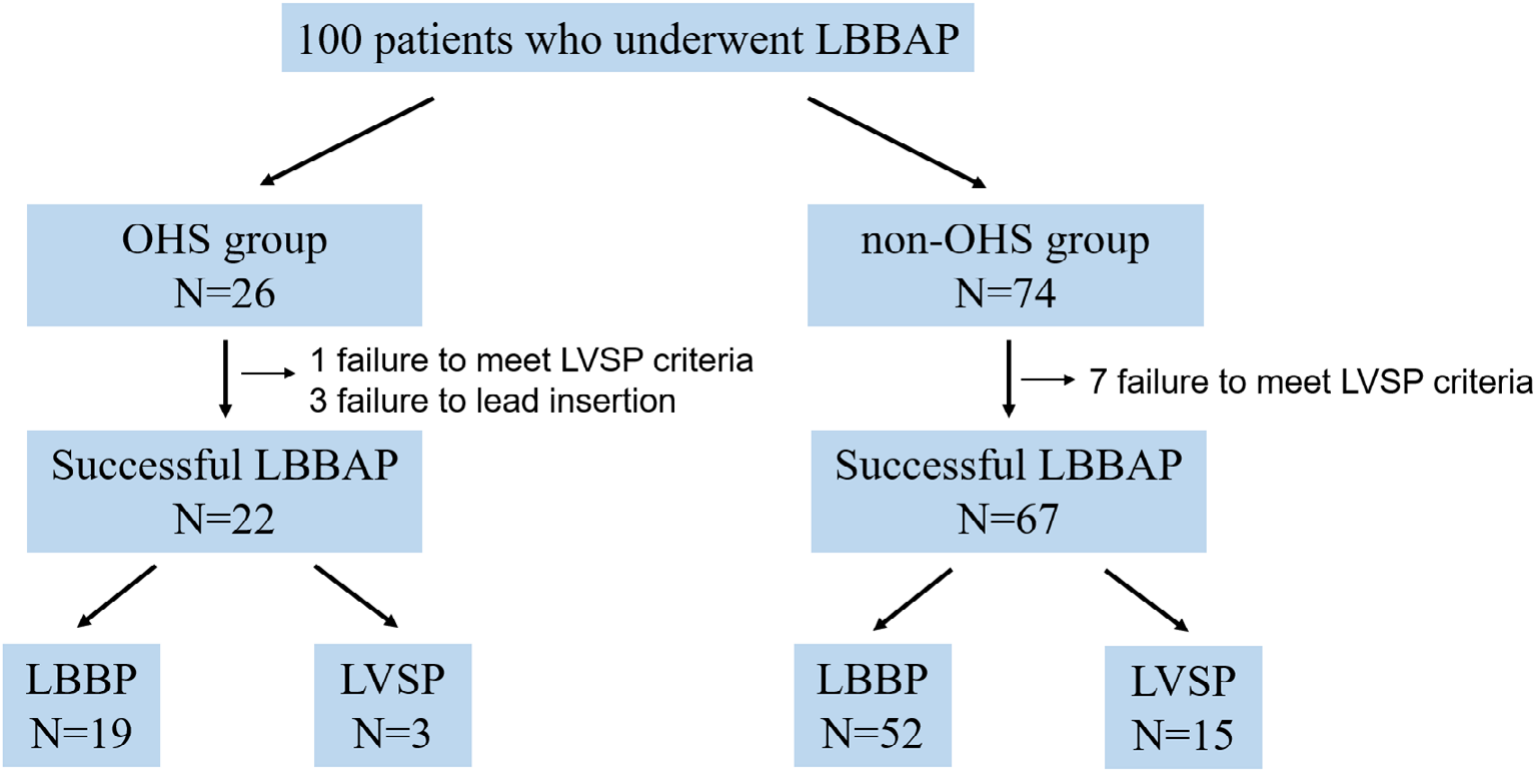
Study protocol. LBBAP, left bundle branch area pacing; LBBP, left bundle branch pacing; LVSP, left ventricular septal pacing.

One hundred patients were analyzed (Figure 1). The study population was divided into two groups based on a history of OHS. Twenty‐six and 74 patients were included in the OHS and non‐OHS group, respectively. LBBAP was successfully achieved in 22 of the 26 (84.6%) patients in the OHS group and in 67 of the 74 (90.5%) patients in the non‐OHS group. The reasons for unsuccessful LBBAP were as follows: In three cases from the OHS group, the lead was slightly inserted into the ventricular septum under fluoroscopic guidance; however, no late R‐wave was observed in V1, and the QRS waveform differed from that during RV pacing prior to screwing, suggesting deep septal pacing. In one case from the OHS group and seven cases from the non‐OHS group, screwing itself was challenging, and the pacing waveform showed no change compared to RV pacing prior to screwing.

The OHS group included 12 cases with valve surgeries (aortic valve replacement [AVR], 2; mitral valve replacement [MVR], 2; mitral annulus plasty [MAP], 3; tricuspid valve replacement [TVR], 1; tricuspid annular plasty [TAP], 1; MVR + TAP, 3), 5 cases with coronary artery bypass grafting (CABG), 1 case of AVR + CABG, 1 case of MAP + CABG, 2 cases of aortic surgeries, and 1 atrial septal defect closure. Only one case underwent interventricular septal incision as part of LV reconstruction, whereas no septal incisions were performed in the other cases. For valve replacement or repair, only annular procedures were performed.

### 3.1 Baseline characteristics

The patient characteristics of the full population and a comparison of both groups are shown in Table 1. No significant clinical differences in the comorbidities, pacing indications, baseline electrograms, or echocardiographic findings were found between the groups. The number of patients with coronary artery disease was significantly higher in the OHS group, which is likely due to the inclusion of six patients who underwent CABG. One case of anteroseptal myocardial infarction with septal wall thinning was present in both groups. The number of patients with non‐ischemic cardiomyopathy included two cases in the OHS group (dilated cardiomyopathy and cardiac sarcoidosis) and eight cases in the non‐OHS group (amyloidosis, 3; dilated cardiomyopathy, 1; muscular dystrophy, 1; unknown etiology, 3), with no significant difference between the groups.

**TABLE 1:** Patient characteristics.

	OHS group (*n* = 22)	Non‐OHS group (*n* = 67)	*p* value
Age, years	76.1 ± 9.0	75.2 ± 11.9	0.743
Male	15 (68.2%)	33 (49.3%)	0.122
Hypertension	5 (22.7%)	27 (40.3%)	0.136
Diabetes	5 (22.7%)	7 (10.4%)	0.143
Atrial fibrillation			
Paroxysmal	4 (18.2%)	6 (9.0%)	0.234
Persistent	6 (27.3%)	10 (14.9%)	0.191
Prior AF ablation	0 (0.0%)	6 (9.0%)	0.146
Coronary artery disease	6 (27.3%)	4 (6.0%)	0.006
Non ischemic heart disease	2 (9.1%)	8 (11.9%)	0.713
Pacing indication			
Sinus node dysfunction	9 (40.9%)	16 (23.9%)	0.123
Atrioventricular block	11 (50.0%)	36 (53.7%)	0.761
Brady AF	0 (0.0%)	2 (3.0%)	0.412
CRT	2 (9.1%)	10 (14.9%)	0.487
lead dysfunction	0 (0.0%)	1 (1.5%)	0.564
AV node ablation	0 (0.0%)	2 (3.0%)	0.412
Baseline electrocardiogram			
QRS duration, ms	134.8 ± 37.4	123.8 ± 29.3	0.159
QRS complex normal	8 (36.4%)	32 (47.8%)	0.351
RBBB	6 (27.3%)	16 (23.9%)	0.749
LBBB	2 (9.1%)	7 (10.4%)	0.855
IVCD	0 (0.0%)	2 (3.0%)	0.412
Escape	4 (18.2%)	7 (10.4%)	0.339
Paced	2 (9.1%)	3 (4.5%)	0.415
Baseline echocardiography			
LVEDD, mm	46.9 ± 10.0	44.7 ± 6.8	0.239
LVEF, %	52.7 ± 15.7	58.0 ± 15.2	0.169
IVS, mm	11.6 ± 1.8	11.6 ± 2.7	0.999

Abbreviations: AF, atrial fibrillation; CRT, cardiac resynchronization therapy; IVCD, intraventricular conduction disturbance; IVS, interventricular septal; LBBB, left bundle branch block; LVEDD, left ventricular end‐diastolic diameter; LVEF, left ventricular ejection fraction; RBBB, right bundle branch block.

Twelve cases meeting the indications of CRT were included. In the OHS group, two cases were identified: one involved the addition of an LBBAP lead because of nonresponse to a CRT defibrillator (CRTD), and the other was a CRTD upgrade following abdominal pacemaker (PM) implantation. In the non‐OHS group, 10 cases were included: three with LBB block and LVEF <35% underwent CRT PM (CRTP) implantation, five with atrioventricular block and LVEF 36%–50% underwent CRTP implantation, and two underwent CRTP upgrade from a previous PM implantation.

### 3.2 Pacing parameters

The pacing parameters for both groups are listed in Table 2. LBBP was successfully achieved in 19 cases (86.4%) in the OHS group and 52 cases (77.6%) in the non‐OHS group, with no significant difference between the two groups (*p =* 0.375).

**TABLE 2:** Pacing parameters at the implant.

	OHS group (*n* = 22)	Non‐OHS group (*n* = 67)	*p* value
LBBP	19 (86.4%)	52 (77.6%)	0.375
LVSP	3 (13.6%)	15 (22.4%)	0.375
LVAT in lead V6, ms	73.6 ± 13.3	75.6 ± 12.1	0.522
V_6_‐V_1_ interpeak interval, ms	43.5 ± 11.3	36.8 ± 11.4	0.019
Capture threshold, V/0.4 ms	0.68 ± 0.34	0.60 ± 0.21	0.195
R‐wave amplitude, mV	12.0 ± 4.7	11.5 ± 5.4	0.733
Impedance, ohms	672.8 ± 101.3	680.3 ± 132.7	0.809
Paced QRS duration, ms	131.3 ± 14.6	121.1 ± 12.3	0.002

Abbreviations: LBBP, left bundle branch pacing; LVAT, left ventricular activation time; LVSP, left ventricular septal pacing.

The LVAT in lead V6 was 73.6 ± 13.3 ms in the OHS group versus 75.6 ± 12.1 ms in the non‐OHS group (*p =* 0.522). The V_6_‐V_1_ interpeak interval was significantly longer in the OHS group (43.5 ± 11.3 ms) compared to the non‐OHS group (36.8 ± 11.4 ms) (*p =* 0.019).

The capture threshold of the LBB area at the implant was similar between the groups 0.68 ± 0.34 V/0.4 ms in the OHS group versus 0.60 ± 0.21 V/0.4 ms in the non‐OHS group (*p =* 0.195). The R‐wave amplitude was similar between the groups (12.0 ± 4.7 versus 11.5 ± 5.4 mV, *p =* 0.733). Additionally, the pacing impedance was similar (672.8 ± 101.3 versus 680.3 ± 132.7 ohms, *p =* 0.809). The paced QRS duration at implant was significantly wider in the OHS group (131.3 ± 14.6 versus 121.1 ± 12.3 msec, *p =* 0.002).

### 3.3 Number of LBBAP lead placements and fluoroscopy time

The number of LBBAP lead placements was significantly higher in the OHS group (3.0 ± 2.1 vs. 2.0 ± 1.4, *p =* 0.017). Fluoroscopy time was similar between the groups (24.2 ± 12.0 vs. 22.2 ± 16.2 min, *p =* 0.588).

### 3.4 Complications and follow‐up

The mean follow‐up time was 16.7 ± 9.8 months (median: 16.5 months, min: 1 and max: 36 months) in the OHS group, and 17.4 ± 12.7 months (median: 18 months, min: 1 and max: 42 months) in the non‐OHS group. Lead displacement, which required lead reimplantation, occurred in one patient in the non‐OHS group. Other acute procedure‐related complications, such as interventricular septum perforation and hematoma, did not occur.

During the follow‐up period, one death occurred in the OHS group (due to end‐stage chronic heart failure), and three deaths occurred in the non‐OHS group (one due to end‐stage heart failure and two from unknown causes).

At the 1‐year follow‐up, including 15 cases (68.2%) in the OHS group and 43 cases (64.2%) in the non‐OHS group, the capture threshold of the LBB area was similar between both groups (0.86 ± 0.19 V/0.4 ms in the OHS group vs. 0.85 ± 0.23 V/0.4 ms in the non‐OHS group, *p =* 0.851). The R‐wave amplitude was similar between the groups (16.5 ± 3.2 vs. 14.4 ± 5.1 mV, *p =* 0.193). Additionally, the pacing impedance was similar (492.1 ± 44.9 vs. 505.4 ± 51.6 ohms, *p =* 0.377) (Table 3). The paced QRS duration was slightly longer at the 1‐year follow‐up than that at implantation in both groups; however, differences between the implantation time‐point and the 1‐year follow‐up were not significantly different in both groups (Table 4).

**TABLE 3:** Pacing parameters at 1 year follow‐up.

	OHS group (*n* = 15)	Non‐OHS group (*n* = 43)	*p* value
Capture threshold, V/0.4 ms	0.86 ± 0.19	0.85 ± 0.23	0.851
R‐wave amplitude, mV	16.5 ± 3.2	14.4 ± 5.1	0.193
Impedance, ohms	492.1 ± 44.9	505.4 ± 51.6	0.377

**TABLE 4:** Paced QRS duration during follow up.

	At implant	6 months	1 year	*p* value (At implant vs. 1 year)
OHS group	131.3 ± 14.6	131.1 ± 15.6	136.1 ± 16.6	0.432
Non‐OHS group	121.1 ± 12.3	122.8 ± 14.8	124.7 ± 13.9	0.215

## 4 DISCUSSION

In the present study, we compared the feasibility and safety of LBBAP in patients with and without OHS. The major findings of this study are described as follows: (1) In patients with a history of OHS, lead insertion for LBBAP was more challenging than in those without such a history. (2) The final success rate of LBBAP was not affected by the presence or absence of a history of OHS, and the lead parameters remained stable over the long‐term in both groups. (3) The QRS duration with LBBAP was longer in patients with a history of OHS than in those without.

LBBAP is widely performed not only as a PM for bradycardia but also as a CRT for patients with cardiomyopathy. Although few studies have investigated LBBAP in patients with certain heart diseases, several have reported the usefulness of LBBAP in patients with prosthetic heart valves.

Guo et al. reported that atrioventricular heart block is a common complication in patients receiving prosthetic valve (PV) implantation, with incidences ranging from 5% to 30% in patients who underwent transcatheter AVR (TAVR), 5% to 10% in patients who underwent surgical AVR, 27% in patients who underwent surgical TVR, and 4.5% to 10.5% in patients who underwent mitral valve repair or MVR.^8^ Some studies have reported that the incidence of PM implantation among patients undergoing surgical or transcatheter PV replacements is between 4% and 17.2%.^9^, ^10^, ^11^

RV apical pacing is the traditional treatment for patients with AV conduction disturbances following PV surgery. However, long‐term RV apical pacing may induce PICM and increase the risk of heart failure and mortality, particularly in patients with atrio‐ventricular conduction diseases after PV surgery who require significant ventricular pacing.^12^, ^13^ Therefore, CSP may be favorable for these patients, to mitigate the risk of PICM. Niu et al. reported procedural and clinical outcomes of CSP post‐TAVR.^14^ In that study, the HBP group had a significantly lower implant success rate, higher capture threshold, and lower R‐wave amplitude than did the LBBP and RVP groups (both *p* < 0.01), and during a mean follow‐up of 15.0 ± 9.1 months, the LBBP group had significantly higher LV ejection fraction (LVEF) (54.9 ± 6.7% vs. 48.9 ± 9.1%, *p* < 0.05) and smaller LV end‐diastolic diameter (49.7 ± 5.6 mm vs. 55.0 ± 7.7 mm, *p* < 0.05) than that of the RVP group. Another study reported that CSP was safe and feasible in patients with a PV, with a significantly higher success rate and superior lead parameters with LBBAP than with HBP, particularly in post‐TAVR patients.^15^ Therefore, the LBBAP is a favorable option for PM implantation in patients with PVs, particularly in those with LV dysfunction.

Although several clinical studies have explored LBBAP in patients with PVs, LBBAP in patients with a history of OHS has not been well described. In the present study, the success rates of the LBBAP and LBBP were found to be comparable between the OHS and non‐OHS groups. The lead parameters at the time of implantation and during the 1‐year follow‐up were favorable and remained stable in both groups. These findings demonstrated that LBBAP can be successfully performed, regardless of a history of OHS.

However, the number of successful LBBAP lead placements was significantly greater in the OHS group. The final success rate of LBBAP was slightly lower in the OHS group, suggesting that the procedure may be more challenging in patients with a history of OHS. One possible reason for this difficulty is fibrosis of the interventricular septal myocardium, which is associated with surgical trauma.

Furthermore, focusing specifically on unsuccessful cases, lead insertion was possible in all seven cases of the non‐OHS group, but they failed to meet the LVSP criteria. In contrast, lead insertion was not possible in three out of four cases in the OHS group. This suggests that fibrosis of the interventricular septum is more likely to occur in the OHS group.

The fibrosis observed in the ventricular septum after OHS is likely influenced by several factors. First, ischemia–reperfusion injury, which occurs during the restoration of blood flow after aortic cross‐clamping, triggers inflammation and fibrosis in the heart muscle. This injury activates inflammatory pathways that contribute to tissue remodeling. In addition, inflammatory cytokines released during surgery, such as those seen in the systemic inflammatory response syndrome, play a significant role in promoting fibrosis by stimulating fibroblast activity and collagen deposition.^16^

Moreover, ischemic injury to the myocardium during surgery leads to necrosis and subsequent scar tissue formation, a process that involves activation of the renin‐angiotensin‐aldosterone system and the sympathetic nervous system.^17^ These neurohormonal responses contribute to maladaptive remodeling of the heart tissue, commonly observed in patients with a history of cardiac surgery. Thus, the fibrosis in the ventricular septum after OHS may result from a combination of factors: ischemic damage, inflammatory responses, and neurohormonal activation. In this study, among the 22 cases in the OHS group, 4 underwent off‐pump CABG and did not require cardiopulmonary bypass. Although ischemia–reperfusion injury does not occur in off‐pump procedures, some cases experience temporary hemodynamic instability because of cardiac displacement maneuver. This may increase the likelihood of ischemic injury compared to cases where cardioplegia was administered effectively during on‐pump procedures.

In addition, the QRS duration during LBBAP was wider in the OHS group, potentially because of baseline characteristics, such as prolonged QRS duration, low LVEF, and LV dilatation. The LVAT in lead V6 was similar between the groups, but the V_6_‐V_1_ interpeak interval was longer in the OHS group. The V_6_‐V_1_ interpeak interval reflects the conductivity of the interventricular septum, suggesting conduction disturbances in the septum in patients with a history of OHS. Consequently, the QRS duration during LBBAP tended to be wider in the OHS group.

The QRS duration remained stable in both groups during the 1‐year follow‐up period, suggesting that physiological pacing was maintained over the long‐term.

### 4.1 Limitations

This study was limited by its retrospective single‐center design and relatively small sample size.

In addition, this study used only the Medtronic flexible catheter sheath (C315HIS). A stiffer catheter sheath, developed by other manufacturers, could potentially increase the success rate and make lead insertion easilier.

Furthermore, preoperative cardiac magnetic resonance imaging was performed in only a few cases, with late gadolinium enhancement in the interventricular septum observed in just one case. As a result, morphological and histological evaluations of myocardial fibrosis were not sufficiently conducted in this study.

Further randomized studies with larger sample sizes are needed to confirm the feasibility and safety of using LBBAP in patients with a history of OHS.

## 5 CONCLUSIONS

LBBAP lead placement in patients with a history of OHS presented a slightly higher level of technical difficulty, but LBBAP was found to be both feasible and safe, regardless of a history of OHS.

## FUNDING INFORMATION

This study did not receive any specific grants from funding agencies in the public, commercial, or nonprofit sectors.

## CONFLICT OF INTEREST STATEMENT

The authors declare no conflicts of interest.

## CONSENT

All patients provided written informed consent.

## ETHICS STATEMENT

The protocol was approved by the institutional review board of Saku Central Hospital (R202410‐01). The research reported in this study adhered to the guidelines of the Declaration of Helsinki (revised in 2013).

## Data Availability

Data from this study are available from the corresponding author upon reasonable request.
